# Drinking Pipes and Nipple Drinkers in Pig Abattoir Lairage Pens—A Source of Zoonotic Pathogens as a Hazard to Meat Safety

**DOI:** 10.3390/microorganisms11102554

**Published:** 2023-10-13

**Authors:** Celine Buder, Diana Meemken, Roland Fürstenberg, Susann Langforth, Alina Kirse, Nina Langkabel

**Affiliations:** 1 Working Group Meat Hygiene, Institute of Food Safety and Food Hygiene, School of Veterinary Medicine, Freie Universität Berlin, 14163 Berlin, Germany; celine.buder@fu-berlin.de (C.B.); roland.fuerstenberg@fu-berlin.de (R.F.); susann.langforth@fu-berlin.de (S.L.); nina.langkabel@fu-berlin.de (N.L.); 2 Veterinary Centre for Resistance Research, School of Veterinary Medicine, Freie Universität Berlin, 14163 Berlin, Germany; 3 Department of Biometry, Epidemiology and Information Processing, WHO Collaborating Centre for Research and Training for Health at the Human-Animal-Environment Interface, University of Veterinary Medicine Hannover, 30559 Hannover, Germany; alina.kirse@tiho-hannover.de

**Keywords:** drinking water system, biofilm, swine, slaughterhouse, *Pseudomonas*, *Salmonella*, *Listeria monocytogenes*, *Yersinia enterocolitica*, methicillin-resistant *Staphylococcus aureus*

## Abstract

The water distribution system in the lairage pens of abattoirs could act as a route of contamination for produced meat. In this study, biofilm formation and the occurrence of specific pathogens in drinking equipment was investigated in different lairage pens in a German commercial pig abattoir. Samples of the water and the drinkers in different locations were microbiologically cultivated and examined. After new drinking equipment had been installed for one month, three months and five years, biofilm formation was detectable, and retrograde growth from the nipple drinkers was seen up to the connection with the main water distribution system. In particular, *Enterobacteriaceae* and *Pseudomonas* spp. were found in all samplings of the nipple drinkers. Zoonotic pathogens, *Salmonella*, pathogenic *Yersinia enterocolitica* and methicillin-resistant *Staphylococcus aureus*, were also isolated from the nipple drinkers, while *Listeria monocytogenes* was not detected via microbial cultivation methods in any of the samples. Since the pigs take the contaminated nipple drinkers into their mouths to drink, or drink contaminated water containing the pathogens, transmission and even infection of the pigs in the lairage can be assumed. This could consequently lead to contamination or cross-contamination of the meat during slaughter and processing and to a public health risk.

## 1. Introduction

Biofilms are complex matrices (polysaccharides, nucleic acids, proteins) with microorganisms, and these matrices can serve to protect the microorganisms from external influences [1]. A biofilm can contain bacteria of different species or of just one species [2]. As a ubiquitous phenomenon, biofilms occur on various surfaces, both biotic (e.g., mucosal cell tissue or wounds) and abiotic (e.g., stainless steel or polyvinyl chloride). As described for *Pseudomonas aeruginosa*, biofilm formation begins with microbial attachment to a surface—which, in the food processing or abattoir environment, is frequently stainless steel (often instruments)—proceeds to form microcolonies and finally, ends with maturation [3], which is possible because of the biofilm’s microbial structures [4].

Biofilms are highly relevant in terms of food safety, and the risk of contamination of the food chain could increase if animals ingest bacteria before slaughter. It takes two hours for pigs to become infected with *Salmonella (S.) enterica* from contaminated lairage pens, and for the pathogen to be detected after slaughter in different organs, i.e., lymph nodes and ileum sections [5]. Studies on the microbial content of drinking water systems have shown that biofilms can exist in this environment [6], and the presence of biofilms in drinking water systems in piglet nurseries was proven [7]. In abattoir lairage pens, the supply of drinking water to the animals is a legal requirement [8]. This water system and the water itself could act as a route of infection if bacteria occur in the water distribution system and are ingested by the pigs. The drinking pipes and installed nipple drinkers in lairage pens of pig abattoirs as a possible source of pathogens and antimicrobial resistance have not yet been researched, but a study proved biofilm can form in water hoses in a meat processing environment [9]. Therefore, there is a lack of peer-reviewed information on the existence and consistency of biofilm formation in the drinking systems in pig lairage pens. In comparison to piglet nurseries with positive test results regarding biofilms, the drinking water systems in abattoirs are not used to administer medication or supplements, such as vitamins. For that reason, a lower biofilm occurrence in drinkers in pig lairages than in piglet nurseries could be assumed.

In addition to various other bacteria, *Pseudomonas (P.) aeruginosa* [10], *Salmonella* [11], *Listeria (L.) monocytogenes* [12], *Yersinia (Y.) enterocolitica* [13] and *Staphylococcus* spp. [14,15] including methicillin-resistant *Staphylococcus aureus* (MRSA) [16] can be frequently isolated from biofilms.

*Pseudomonas aeruginosa* is a human pathogenic, Gram-negative bacterium with a ubiquitous presence in many places (e.g., soil, plants, tissue, air, surfaces), particularly common in water-related or hydrous material (pipes, drains and water itself). *Pseudomonas* spp. are major biofilm builders and contribute to its formation in environmentally challenging places, e.g., faucets, drains and showerheads [10,17].

*Salmonella*, a Gram-negative bacterium, mainly occurs in the gastrointestinal tract of humans and pigs [18,19,20]. Some of the most common serovars in humans are *S. Typhimurium* and *S.* Derby, which can cause severe infections and are commonly found in pigs, with *S*. Derby being the most common serovar on pig carcasses [21]. Contamination of meat can be caused by direct contact (with the gastrointestinal tract, faeces, skin, lymph nodes during processing or evisceration) or indirect transmission (via knives and abattoir staff) [22,23,24,25,26]. *Salmonella*-containing biofilms occur on non-organic materials, such as plastic, rubber, cement and stainless steel, especially on the latter, which is frequently used in abattoirs [11,27,28].

*L. monocytogenes*, a Gram-positive bacterium, develops or occurs in biofilms in drains, floors or on contact surfaces, especially when water accumulates [12]. *Listeria* spp. are known to attach onto different kinds of surfaces and to survive different kinds of stressors [12,29,30]. Studies show that *Listeria* spp. occur on pig farms, in abattoirs and in the food processing industry being isolated from the skin or faeces of pigs [31,32,33]. Therefore, infected pigs can transfer *Listeria* spp. in the food chain and cause contamination and recurrent infiltration of the surroundings of abattoirs, as well as cutting and processing plants [29].

*Y. enterocolitica*, a Gram-negative bacterium, causes severe human infections as a foodborne disease originating mainly from pigs that are asymptomatic carriers of *Y. enterocolitica* [13,15,34,35,36]. Proven sources are pig tonsils, tongue, intestine and faeces, from which the pathogen can cross-contaminate knives and other parts of the processing equipment, as well as the carcasses [37]. Biofilm-forming ability has been proven under conventional conditions [13].

*Staphylococcus aureus*, a Gram-positive bacterium, can be a contaminant of meat products [15]. An important aspect is the antibiotic resistance of *Staphylococcus aureus*, for example in MRSA, which causes great problems in treating patients and controlling bacteria [38]. The ability to produce multi-layered biofilm in wide ranges of temperature, pH levels and sodium chloride concentrations makes it possible for this pathogen to survive in dry and stressful environments [39,40,41].

Given the abovementioned dearth of scientific information on biofilms in drinking systems in pig lairages, in this study, the biofilms in drinking water pipes and nipple drinkers in lairage pens in a commercial German pig abattoir were investigated after the equipment had been installed and used for different periods of time. The main aim was to analyse, according to the different durations the equipment had been installed, the presence of biofilms in the drinking water equipment of pig lairage pens. Additionally, the study aimed to determine whether these biofilms are reservoirs of *Pseudomonas* spp., *Salmonella*, *L. monocytogenes*, pathogenic *Y. enterocolitica* or MRSA.

## 2. Materials and Methods

### 2.1. The Water Distribution System

**Figure 1:**
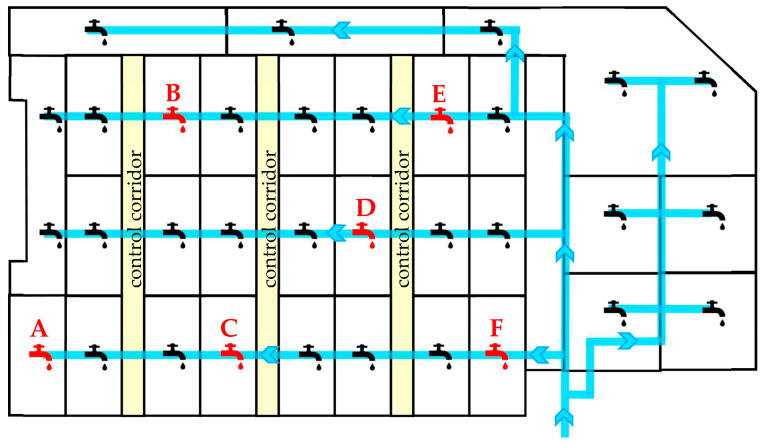
Schematic layout of the lairage showing control corridors (yellow) and pens (black lines). Blue lines show the main water distribution system; arrows symbolise the direction of waterflow; drinker symbols show each drinker in the lairage pens; the drinkers sampled in this study are marked in red (**A**–**F**); control corridors are used for animal inspection and by the abattoir staff.

For the investigations, six lairage pens in a commercial German pig abattoir were selected, each with a holding capacity of 17 to 19 pigs. The abattoir had a slaughter capacity of approximately 2900 pigs/day. Each lairage pen had one drinker, so those were named Drinker A to F. The location of these six pens was selected to represent a homogeneous distribution in terms of size and occupancy, so a similar frequency of use was assumed. To also show heterogeneous characteristics, the distances from the source of water (which impact water speed and pressure), the dead ends in the water system and the accessibility of the pens (central and marginal) were considered in selecting the pens. Control corridors were used by abattoir staff and veterinarians for animal inspections (Figure 1).

The connections between drinkers and the main water distribution system were joint elements, similar to those used in domestic garden watering systems, made of plastic. Each commercially supplied drinker consisted of an 88 cm long stainless-steel pipe, which ended in a 4-fold pipe cross, each end finishing with a nipple drinker, resulting in four nipple drinkers per drinker (Hölscher & Leuschner, Emsbühren, Germany). The water fed into the drinkers consisted of 75% artesian water from a well owned by the abattoir company and 25% tap water from the public water supply system. The quality of the water mix for final use in the abattoir was tested by the abattoir regularly, in accordance with legal regulations.

### 2.2. Sampling

**Figure 2:**
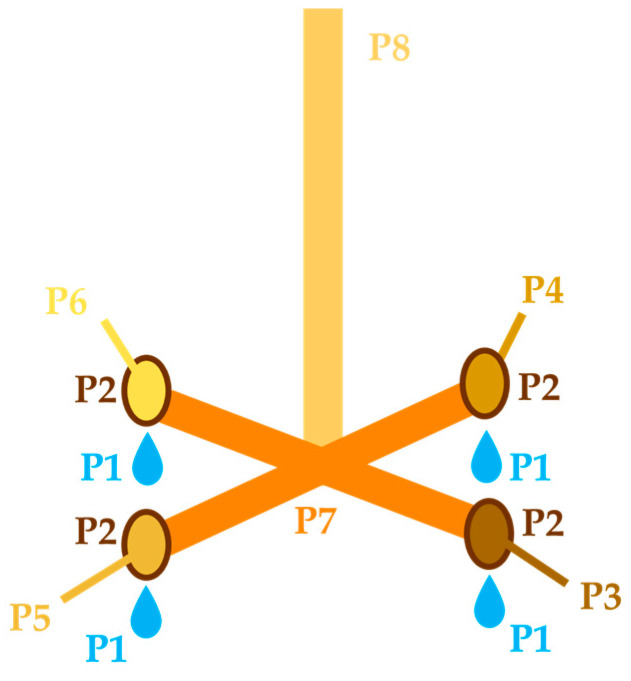
Schematic overview of the drinker and the sample types (P1–P8).

From each drinker, eight samples were taken (Figure 2). Firstly, a pool sample from all four nipples of the water in each drinker (P1) was obtained, in order to assess the hygienic status of the ingested water. Each nipple was released individually by hand (covered with new, disposable gloves). The empty, sterile, plastic sample tube (Sarstedt AG & Co. KG, Nümbrecht, Germany) was filled with approximately 25 mL water from each nipple, resulting in a 100 mL sample, P1. Afterwards, the drinkers were disassembled, held up so contact with the floor was avoided, and samples were taken as follows. For all swab sampling, dry and individually packed swabs (Sarstedt AG & Co. KG, Nümbrecht, Germany) were utilised, and care was taken to break off and discard each swab stick that had touched a new, disposable sampling glove. A pooled swab sample from all four nipple drinkers (P2) was taken. This was done to evaluate the superficial hygienic status of the nipple drinkers that are taken into the pigs’ mouths while they drink. Each nipple drinker was sampled with two swabs by swiping the outer front part of the nipple drinker, where the pig has direct contact with the material. More precisely, for each nipple drinker, an initial swab was used to swipe the still-wet nipple drinker (wet because we had just taken P1 water samples, described above) from the frontal aspect of the nipple drinker, including the nipple tip where the water emerges. The second nipple drinker swab was used to swipe the exact same area once again. The eight swabs per drinker were placed together in one sample tube to produce P2. Afterwards, each nipple drinker was disassembled individually and separately placed into a sterile sample tube (P3, P4, P5 and P6). After the removal of the nipple drinkers, to assess if retrograde microbial growth could be detected from the nipple drinkers to the inner lower parts of the pipes, a pooled swab sample of the inner pipe surfaces was taken (P7). The inner surface of the pipe was swabbed in a circular movement from the pipe cross to each opening, covering the whole inner area. This procedure was repeated for all four parts of the pipe cross with two dry swabs per section, one after the other. All eight swabs were placed together in one empty, sterile sample tube to produce P7. The last sample (P8) was taken from the upper pipe section, right before the connection to the main water distribution system, to examine if any retrograde growth was present up to the top end of the drinker. Two dry swabs were swiped in sequence inside the upper pipe section in sequence, and with circular movements, from the top end to the opening, and placed together in one empty, sterile sample tube to produce P8. After sampling, the six drinkers in the lairage pens were replaced by new drinkers sourced from the same manufacturer and of the same materials and dimensions.

The plan of sampling for this study was divided into three parts. The first part was the sampling of the drinking pipes and nipples installed to date (samples P1–P8, see above). The drinking equipment had been installed for approximately five years. After examination of these used drinking pipes and nipples, all examined pens were equipped with new drinkers, which were sampled (P1–P8) after being installed for one month. After this second examination, the drinking equipment was again replaced with new drinkers and used for three months before the third and final sampling (P1–P8) was conducted.

### 2.3. Bacteriological Examination

After collection, the samples were sent via express mail in a cooled box at approximately 7 °C to the laboratory of the Institute of Food Safety and Food Hygiene, Working Group Meat Hygiene at Freie Universität Berlin, Germany, where they arrived in the morning the day after sampling. Examinations based on the respective German standards (DINs) started the day after sampling: quantitative analysis of total aerobic plate count (APC) [42], *Enterobacteriaceae* count (EB) [43] and *Pseudomonas* spp. count [44], and qualitative analysis of *Salmonella* [45], pathogenic *Y. enterocolitica* [46], *L. monocytogenes* [47] and MRSA [48].

The water sample (P1) was directly processed, diluted and applied on the appropriate agar media. For APC, Plate Count Agar (PCA; Th. Geyer GmbH & Co. KG, Renningen, Germany) was incubated for 72 ± 2 h at 30 °C, and for EB, Violet Red Bile Dextrose agar (VRBD; Merck KGgA, Darmstadt, Germany) was incubated for 48 h at 37 °C, following the respective standard procedures for water [49,50]. All other samples were diluted by adding 50 mL buffered peptone water (BPW, Merck KGgA, Darmstadt, Germany) and then cooled in the refrigerator at 6 °C for 30 min. To achieve better solution of the particles visible on the swabs, P2, P7 and P8 were homogenised on a whirl mixer for 30 s, and the individual nipples (P3–P6) were manually shaken for 1 min. Afterwards, 5 mL of the final homogenate of each sample was pipetted into a new sample tube (classified as the original sample or respectively as dilution level 0 (10^0^)), and dilution series in 1:9 ratio with sodium peptone agar (Merck KGgA, Darmstadt, Germany), were created. For P2, P3–P6 and P7, to determine APC and EB, 0.05 mL of each dilution were applied on PCA and VRBD, respectively, following the respective DINs [42,43], and spread with a sterile loop (Sarstedt AG & Co. KG, Nümbrecht, Germany), before incubating as described above. For samples P1 and P8, to determine APC and EB, 0.1 mL of each dilution were applied to the same agars and spread with a sterile spatula (Sarstedt AG & Co. KG, Nümbrecht, Germany) before incubation. For *Pseudomonas* spp., 0.1 mL of dilution levels 0 and 1 were applied to *Pseudomonas* CFC Agar (Oxoid Deutschland GmbH, Wesel, Germany), following the DIN [44], and spread with a sterile spatula before incubating for 40–48 h at 25 °C.

After incubation, all colony-forming units (CFU) per sector on the agar plates were counted. The counts from two consecutive dilution levels were used to obtain a weighted average, and in the case of P1, counts were directly transformed to the logarithm of base 10. For P2–P8, the area was included in the calculation, and afterwards, the counts were logarithmically transformed. For all swab samples, the specific area was included in the calculation of the CFU: P2 = 88.5 cm^2^, P7 = 149.5 cm^2^ and P8 = 70 cm^2^. The nipple drinkers (P3–P6) had an unknown surface area due to complexity of these structures. Therefore, based on DIN EN ISO 18593:2018-10 [51] and DIN 10113-1:2023-02 [52], the area for the calculation was counted as 1, meaning that the bacteria were not counted per cm^2^ but per nipple drinker.

For the qualitative examinations, for pathogenic *Y. enterocolitica*, 0.5 mL of each original homogenate were spread onto *Yersinia* selective medium (CIN) (Oxoid Deutschland GmbH, Wesel, Germany) and incubated for 24 h at 30 °C. For MRSA, 0.1 mL of each original homogenate were spread onto Agar Baird Parker (Oxoid Deutschland GmbH, Wesel, Germany) and Columbia CNA Agar (Oxoid Ltd., Basingstoke, UK) and incubated for 24 h at 37 °C. Another 5 mL of each original homogenate were added into specific pre-enrichment broths for *L. monocytogenes* (Half Fraser Broth; Oxoid Deutschland GmbH, Wesel, Germany), incubated for 24–26 h at 30 °C, and for pathogenic *Y. enterocolitica* (Peptone Sorbitol Bile Broth; Sigma-Aldrich Chemie GmbH, Steinheim, Germany), incubated for 40–48 h at 25 °C. After incubation of the *L. monocytogenes* pre-enrichment, 0.1 mL was transferred into the next enrichment broth, (Fraser selective medium; Oxoid Deutschland GmbH, Wesel, Germany) and incubated for 24 ± 2 h at 37 °C. Additionally, 0.01 mL of the *L. monocytogenes* pre-enrichment culture was spread onto *Listeria* selective agar ALOA (VWR International GmbH, Dresden Germany) and Palcam Agar (Oxoid Deutschland GmbH, Wesel, Germany) and incubated for 48 h at 37 °C. The original homogenate with BPW was incubated as a *Salmonella* pre-enrichment for 16–18 h at 37 °C. Afterwards, 0.1 mL were transferred into Rappaport-Vassiliadis-Soya broth (Merck KGaA, Darmstadt, Germany) and incubated for 24 h at 42 °C, and into Muller-Kaufmann Tetrathionate Novobiocin broth (Merck KGaA, Darmstadt, Germany) incubated for 24 h at 37 °C. Following incubation, 0.01 mL of these enrichment broths were separately spread onto both Brilliant Green Agar (Oxoid Deutschland GmbH, Wesel, Germany) and Rambach-Agar (VWR International GmbH, Dresden, Germany) before incubation for 24 h at 37 °C. Further examination was performed according to the respective DINs [45,46,47,48]. Bacteria that were identified as *Salmonella* were serotyped following the Kauffmann–White–Le Minor scheme [53]. Further identification of the other confirmed bacterial pathogens that were analysed qualitatively was performed by specific polymerase chain reactions (PCR), performed according to Garzetti et al. (2014) [54] for pathogenic *Y. enterocolitica*, Bubert et al. (1999) [55] for *L. monocytogenes*, and Strommenger et al. (2003) [56] and Jonas et al. (2002) [57] for MRSA.

### 2.4. Statistical Analysis

All data was used, i.e., outliers were not excluded from further analyses due to their general plausibility. All original bacteria count data was log-transformed to the base 10 before statistical analyses. Descriptive analyses were conducted for each sampling of the installed drinking equipment in relation to the sample types and for each sample type in relation to the duration the drinking equipment had been installed, respectively. For analyses of variance, the Kruskal–Wallis Test was performed with no further adjustment for multiple testing due to the explorative nature of the investigation. An α-level of ≤0.05 was set as the limit of statistical significance of the individual sample types compared between the three sampling time frames, but not comparing the sample types with one another due to the different sample units. All statistical analyses were conducted using SAS^®^ version 9.4.3 (SAS Institute Inc., Cary, NC, USA).

## 3. Results

While the sampling was performed in the order of time durations as presented in Section 2, i.e., five years, one month and then three months, the results are presented in chronologically ascending order from one month to three months to five years.

### 3.1. Total Aerobic Plate Count (APC)

#### 3.1.1. One-Month-Installed Drinking Equipment

In the water samples (P1), APC ranged from 3.96 to 6.10 log CFU/mL (Table 1, Table S1). For P2, APCs ranged between 6.18 to 7.23 log CFU/cm^2^. For the individual nipple drinkers (P3–P6), the mean APC was above 7.24 log CFU/nipple. APC of the lower pipe samples, P7, ranged from 1.69 to 2.87 log CFU/cm^2^ and from 0.85 to 2.30 log CFU/cm^2^ for the upper pipe sample, P8.

In P2, the drinker with the highest APC per cm^2^ was Drinker E with 7.23 log CFU/cm^2^, followed by Drinker D (6.95 log CFU/cm^2^) and Drinker F (6.94 log CFU/cm^2^). The drinker with the highest APC per nipple drinker was Drinker F with a mean value of 7.89 log CFU/nipple from P3–P6. Drinker B and E had the second highest mean value of APC per nipple drinker (7.73 log CFU/nipple).

#### 3.1.2. Three-Months’ Installed Drinking Equipment

After drinking equipment was installed for three months, APC in the water samples (P1) ranged from 4.00 to 5.09 log CFU/mL. In P2, APC ranged between 5.82 and 7.22 log CFU/cm^2^. The mean APC for P3–P6 ranged from 7.51 to 7.90 log CFU/nipple. P7 showed APC values between 2.84 and 3.94 log CFU/cm^2^ and P8 between 1.43 and 4.63 log CFU/cm^2^; one sample of P8 could not be evaluated.

The water sample of Drinker B, installed for three months, had the highest results for APC per mL with 5.09 log CFU/mL. Drinker F, with 4.88 log CFU/mL, had the second highest result and Drinker E had the third highest (4.47 log CFU/mL). The drinker with the highest APC per cm^2^ was Drinker E, with 7.22 log CFU/cm^2^ in P2, followed by Drinker D (6.95 log CFU/cm^2^ in P2) and Drinker F (6.94 log CFU/cm^2^). Drinker F was the drinker with the highest APC per nipple drinker, with a mean APC of 7.90 log CFU/nipple. Drinker E showed the second highest result (7.68 log CFU/nipple) and Drinker D the third highest (7.66 log CFU/nipple).

#### 3.1.3. Five-Years’ Installed Drinking Equipment

In drinking equipment installed for five years, the APC for P1 ranged from 4.55 to 5.58 log CFU/mL. P2 showed values between 5.61 and 6.20 log CFU/cm^2^, and the mean APC for P3–P6 ranged from 6.97 to 7.81 log CFU/nipple. P7 ranged from 2.78 to 4.33 log CFU/cm^2^. Three samples of P8 from Drinker A, C and E showed no bacterial growth for APC, while the other three had a value of 1.85 log CFU/cm^2^.

In drinking equipment installed for five years, the highest APC determined was in the water (P1) of Drinker D, with 5.58 log CFU/mL. The second highest APC in water was 5.42 log CFU/mL in Drinker B, and Drinker E had the third highest APC in water at 5.08 log CFU/mL. Among the P2 samples from drinking equipment installed for five years, Drinker F showed the highest APC per cm^2^, with 6.20 log CFU/cm^2^. Drinker E showed the second highest APC for P2 (6.04 log CFU/cm^2^) and Drinker B the third highest (5.98 log CFU/cm^2^). The drinker with the highest APC per nipple drinker was Drinker B, with a mean value of 7.81 log CFU/nipple, followed by Drinker F (7.62 log CFU/nipple) and Drinker D (7.54 log CFU/nipple).

#### 3.1.4. Comparison of APCs after Drinking Equipment Had Been Installed for Three Time Durations

**Figure 3:**
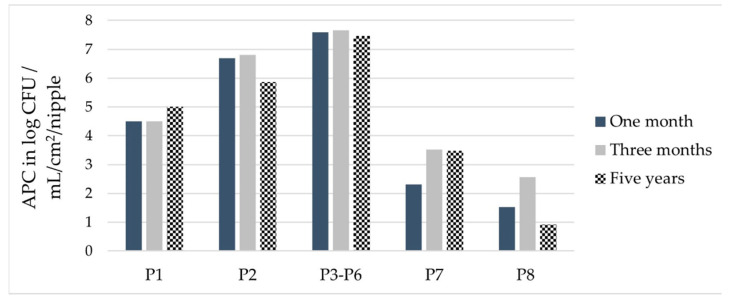
Mean total aerobic plate count (APC) per sample type after the drinking systems had been in use for one and three months and five years; P1: water sample in log CFU/mL; P2: pooled nipple swab sample in log CFU/cm^2^; P3–P6: individual nipple sample in log CFU/nipple; P7: pooled lower pipe section swab sample in log CFU/cm^2^; P8: pooled upper pipe section swab sample in log CFU/cm^2^.

Among the tested durations for which drinkers were installed and used, in the drinking equipment installed for one and three months, 48/48 and 47/48 samples showed bacterial growth, respectively, marking the drinking equipment installed for five years as the only equipment from which bacteria were not able to be cultivated from some samples (these were P8, the upper pipe section). The water sample (P1) had the highest mean APC in the drinking equipment that had been installed for five years (Figure 3), but the differences in median APCs were not statistically significant between the three installation durations (Table A1). In the pooled nipple swab samples (P2), the difference in median APC was statistically significant between the three installation durations. The APC was the lowest after five years’ installation, followed by one month and then three months’ installation. The individual nipple drinkers (P3–P6) showed the same distribution over the three installation durations, with statistically significant different medians. The APCs of P7 were the highest after the drinking equipment was installed for three months. The APCs of P7 determined after the drinking equipment had been installed for five years were nearly the same as those determined after three months, while the lowest APCs were found in the drinking equipment after it had been installed for one month. Although the APC of P8 was notably lower in the drinking equipment installed for five years than for the other two installation durations, the differences in median APCs were not statistically significant between the installation durations.

### 3.2. Enterobacteriaceae Count

EB were found only in the pooled nipple swab samples (P2) or individual nipple drinkers (P3–P6); all other sample types were negative.

In drinking equipment installed for one month, 31.3% (15/48) of the samples were positive, coming from all drinkers except Drinker B. In drinking equipment installed for three months, 27.1% (13/48) of the samples were positive for EB, distributed over all drinkers. In drinking equipment installed for five years, 25.0% (12/48) of the samples were positive for EB. Here, no EB were detected in Drinker A (Table 1, Table S1).

Comparing all three installation durations, the occurrence of samples with EB went from 15 (one month), to 13 (three months), to 12 (five years) out of 48 samples for each time duration. No statistically significant differences in median EB counts between the three installation durations could be observed (Table A1). The highest EB count per cm^2^ occurred in Drinker D after it had been installed for five years (3.85 log CFU/cm^2^). The highest EB count per nipple drinker was registered for Drinker F after it had been installed for one month (mean EB count for the individual nipple drinkers was 3.34 log CFU/nipple).

### 3.3. Pseudomonas spp. Count

#### 3.3.1. One-Month Installed Drinking Equipment

In drinking equipment installed for one month, *Pseudomonas* spp. was detected in 57.1% (24/42) of the samples. Positive samples were the pooled nipple swabs (P2) with 1.99 to 3.33 log CFU/cm^2^ and the individual nipple drinkers (P3–P6) with mean values from 1.85 to 4.53 log CFU/nipple, distributed over all six drinkers. No *Pseudomonas* spp. was detected in the lower or upper pipe sections (P7 and P8) (Table 1, Table S2).

#### 3.3.2. Three-Months’ Installed Drinking Equipment

In drinking equipment installed for three months, *Pseudomonas* spp. was detected in 59.5% (25/42) of the samples, distributed over all six drinkers. Positive samples were the pooled nipple samples (P2) and individual nipple drinkers (P3–P6). P2 samples had *Pseudomonas* spp. counts from 1.35 to 3.56 log CFU/cm^2^ in all six drinkers. In all positive samples of P3–P6, *Pseudomonas* spp. mean values ranged from 0.88 to 5.02 log CFU/nipple (Table 1).

#### 3.3.3. Five-Years’ Installed Drinking Equipment

In drinking equipment installed for five years, *Pseudomonas* spp. was found in 31.0% (13/42) of the samples, but only in P2 and P3–P6. The two positive P2 samples had counts of 0.75 and 3.33 log CFU/cm^2^, and P3–P6 ranged from 0.75 to 2.67 log CFU/nipple (Table 1).

#### 3.3.4. Comparison of *Pseudomonas* spp. Counts after Drinking Equipment Had Been Installed for Three Time Durations

All sample types that harboured *Pseudomonas* spp. were associated with the nipple drinkers (P2, P3–P6). Both P2 and P3–P6 showed the highest counts in the drinking equipment after it had been installed for three months; after one month, lower *Pseudomonas* counts were detected. The differences in median *Pseudomonas* spp. counts for the individual nipples (P3–P6) were statistically significant between the three installation durations (Table A1). The mean *Pseudomonas* spp. count of the four individual nipple samples was lower in the drinking equipment installed for five years compared to the other two installation durations. All drinkers at all three sampling times contained *Pseudomonas* spp. in differing levels, but the organisms were always detectable. The highest percentages of positive samples were found in Drinkers D and F for all three installation durations and sample types (61.9%, 13/21). Drinkers A and B had the fewest positive samples (38.1%, 8/21).

### 3.4. Detection of Other Specific Pathogens

#### 3.4.1. *Salmonella*

In drinking equipment installed for one month, 9.5% (4/42) of the samples were *Salmonella*-positive. These samples were all nipple-associated samples (P2, P3–P6) of Drinker E. In all *Salmonella*-positive samples, the serovar isolated was identified as *S.* Derby. After three months, one *Salmonella*-positive sample, identified as *S*. Typhimurium, was detected in Drinker D (P3–P6). *Salmonella* was not detected from any sample in the drinking equipment after it had been installed for five years (Table 2, Table S2).

#### 3.4.2. *Listeria monocytogenes*

*L. monocytogenes* was not detected in any of the samples (Table 2, Table S2), and neither were any other *Listeria* species.

#### 3.4.3. Pathogenic *Yersinia enterocolitica*

In drinking equipment installed for one month, pathogenic *Y. enterocolitica* was found in two individual nipple drinkers and one pooled nipple sample (P2) of Drinker D, (7.1%; 3/42). All other drinkers and samples were negative for this organism in this equipment. In drinking equipment installed for three months, one pooled nipple swab sample, P2, from Drinker A harboured pathogenic *Y. enterocolitica* (2.4%; 1/42). In drinking equipment installed for five years, no pathogenic *Y. enterocolitica* was found (Table 2, Table S2).

#### 3.4.4. Methicillin-Resistant *Staphylococcus aureus* (MRSA)

In drinking equipment installed for one month, MRSA was detected in 7.1% (3/42) of the samples. All positive samples were individual nipple drinkers of three different drinkers: Drinkers B, D and F. In the drinking equipment installed for this time period, no MRSA was found in the sample types P2, P7 or P8. In drinking equipment installed for three months, no MRSA was detected. In drinking equipment installed for five years, 26.2% (11/42) of the samples were positive for MRSA. All these positive samples were nipple-associated (P2, P3–P6) from Drinkers B, C, E and F, while MRSA was not isolated from any of the lower or upper pipe sections (Table 2, Table S2).

### 3.5. Drinker Evaluation

**Figure 4:**
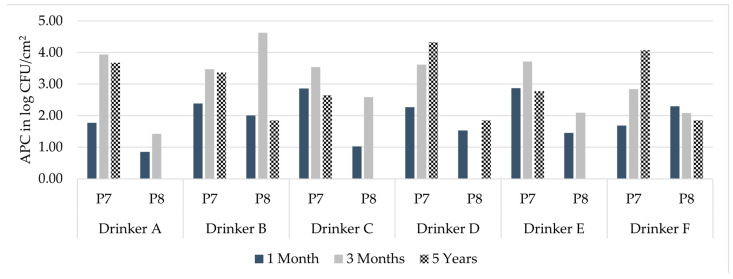
Total aerobic plate count (APC) of the sample types P7 and P8 of the six drinkers after the drinking systems had been installed for one and three months and five years; P7: pooled lower pipe section swab sample; P8: pooled upper pipe section swab sample.

As previously described, APCs were at detectable levels in nearly all samples of the three installation durations. To demonstrate the actual occurrence of biofilm formation without any pathogens that could appear due to direct contact between pigs and drinking equipment, the pipe samples need to be evaluated specifically (Figure 4). The sample of the lower inner pipe surface (P7) and the upper inner pipe surface (P8) demonstrated biofilm formation occurred in these areas, as the APC bacteria could not have come from pigs contacting these parts of the drinking equipment. Moreover, these APC data allow additional comparison of the biofilm formation in the separate drinkers. In drinking equipment installed for one month, Drinker E showed the highest APCs for P7, followed by Drinker C and Drinker B (Table 1). Drinker F showed the highest APC results for P8 in drinking equipment that had been installed for one month, followed by Drinker B and Drinker D. The lowest results for both samples P7 and P8 were found in Drinker A. In drinking equipment installed for three months, Drinker A showed the highest APC results for P7, Drinker E the second highest and Drinker D the third highest. P8 had the highest APCs in Drinker B, then Drinker C and Drinker E. The lowest APCs in drinking equipment installed for three months were seen in Drinker F for P7 and Drinker A for P8. In drinking equipment installed for five years, the highest APC for P7 was identified in Drinker D, followed by Drinker F and Drinker A. The lowest APC for P7 was in Drinker C. P8 had the same level in all APC-positive samples, seen in Drinkers B, D and E. In other drinkers (A, C and E), APCs were not detectable.

EB was detected only in the nipple-associated samples, P2 and P3–P6, with the highest count in Drinker F, after it had been installed for one month (Table 1). Drinker D had the second highest and Drinker E the third highest EB counts. In drinking equipment installed for three months, the highest EB counts were seen in Drinker D, followed by Drinker B and Drinker A and F. After Drinker D had been installed for five years, it showed the highest EB counts, with Drinker C having the second highest and Drinker B the third highest.

*Pseudomonas* spp. were also only seen in the sample types P2 and P3–P6. In drinking equipment installed for one month, Drinker F showed the highest *Pseudomonas* spp. count, Drinker E having the second highest and Drinker C the third highest (Table 1). In drinking equipment installed for three months, Drinker C had the highest *Pseudomonas* spp. count, followed by Drinker F and Drinker B. In drinking equipment installed for five years, Drinker D had the highest *Pseudomonas* spp. count, while Drinker C had the second highest and Drinker E the third highest.

**Figure 5:**
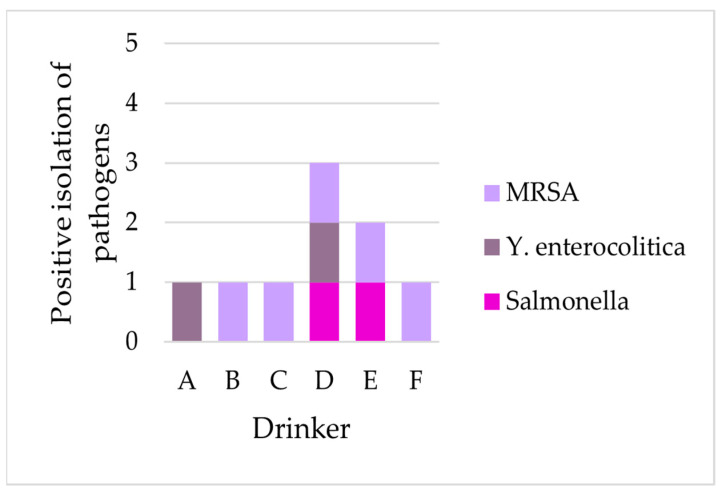
Number of samples with positive isolation of the tested specific pathogens in all three installation durations per drinker A to F; *Y. enterocolitica*—pathogenic *Yersinia enterocolitica*; MRSA—methicillin-resistant *Staphylococcus aureus*.

*Salmonella* occurred only in Drinker D and Drinker E, while pathogenic *Yersinia enterocolitica* could only be isolated from Drinker A and D over all three installation durations studied. MRSA was found in all drinkers, except for Drinker A (Table 2, Figure 5).

## 4. Discussion

### 4.1. Distribution of Bacterial Content in the Sample Types

Differences were observed in counts of APC, EB and *Pseudomonas* spp., as well as in the occurrence of specific pathogens. It should be noted that the elevated APCs determined in the water samples do not allow any conclusion to be drawn about the drinking water quality. The official water analyses provided by the abattoir showed that the supplied water going into the drinking system complied with the quality required by German national drinking water standards [58]. However, the water samples in this study were deliberately taken at the end of the drinking system, specifically from the nipples where the pigs drink, to demonstrate the extent to which microbial contamination is present during the drinking process. It can be concluded that the pigs in the lairage pens can ingest contaminated water during drinking, as the nipples, which are frequently surrounded by a biofilm or film of saliva, feed and faeces particles, produced high APCs and harboured three of four examined specific pathogens. Even though EB were not detected in any of the water samples, there is a chance water was actually contaminated with EB or the other specific pathogens, because the amount of water tested was comparatively small.

Since the evidence suggests that the regularly tested supplied water was not the origin of contamination and biofilm formation, retrograde growth of biofilm seems to be plausible. APCs were at detectable levels in every sample type, and the APCs followed a clear decrease from the nipples with direct animal contact to the upper pipe section, marking the characteristics of retrograde growth, as bacteria have to grow from the source upwards and against the water flow. Although the length to which bacteria grew in a retrograde fashion was not measured directly, by comparing the different sample types with their locations, growth of at least 88 cm can be assumed, as this is the length from the pipe cross to the upper sampled pipe section. EB, *Pseudomonas* spp., *Salmonella*, pathogenic *Y. enterocolitica* and MRSA were obtained from the pooled swabs of the nipples (P2), as well as from the individual nipple drinkers (P3–P6), allowing contamination of water as it flowed through the nipples and was consumed by the pigs.

### 4.2. Biofilm Formation

In terms of APC, which marks universal biofilm formation [59,60], a general APC increase in the drinking equipment was noticeable according to the duration of installation and use, in the drinkers and sample types. *Pseudomonas* spp., as a general biofilm builder [10], had proliferated to the greatest levels after one and three months, which also shows that the biofilm formation peaked in this time period. The drinking equipment that had been installed for five years had the lowest APC and *Pseudomonas* spp. levels in most of the samples. The difference in median APC for P2 and P8 was statistically significant between the three installation durations. APCs were lower in the drinking equipment installed for five years compared to in equipment installed for one and three months. The individual nipple samples showed statistically significant differences in median *Pseudomonas* spp. levels between the installation durations. Overall, the drinking equipment installed for five years harboured lower *Pseudomonas* spp. counts.

These observations can be explained by general biofilm formation characteristics. Biofilm formation consists of recognisable stages: conditioning, attachment and growth, metabolism and dispersion, and the final level of colonisation, meaning the end of the biofilm formation in this location. At this point, new areas for biofilm formation are established by the bacteria returning into a motile form, while the biofilm in the original location stays in a stable form. At this point, the biofilm has finished its metabolism in this exact location and expands to others [61,62]. Usually, this stage is reached after 12 to 18 months [63]. Furthermore, the biofilm does not have to be a continuous layer. Instead, it can consist of many clusters ranging in size and can become round or extended under suitable hydrodynamic conditions, depending on the form of the waterflow [61,64]. The existing waterflow form is crucial for the detachment of biofilm clusters, but in general, detachment is only possible if the external shear forces, in this case caused by the hydrodynamic flow, surpass the internal cohesive strength of the biofilm matrix [64].

While it has been confirmed that microorganisms coming from biofilms attached to inner pipe surfaces can appear in drinking water [6,63], a study has also shown that only 5% of the bacteria from these biofilms are found in the water, meaning that 95% are still attached to the surface [65]. Therefore, the risk of pigs becoming infected by specific pathogens found in the nipple area originating from the water may be low, but must be considered. Additionally, only some of the bacteria may be detectable by classical microbiological methods like cultivation. Some bacteria transition into a non-culturable state [66], and therefore, these would not have been detected by the microbiological methods used in this study. To visualise the full scope of bacteria present, other microbiological methods could be useful, e.g., ultrafiltration of a larger amount of water that flows through the drinking equipment and analysing the sediments with PCR [67,68,69]. To visualise the actual biofilm, an electron microscopic scan could be used [70]. This could be interesting for further investigations, as this study was to obtain an initial overview of the state of biofilm formation in the drinking equipment of a pig abattoir.

While the results for APC and *Pseudomonas* spp. show a tendency for development and content of biofilm, the same assumptions cannot be made for EB. While the EB counts after one month were higher than after three months’ installation, in drinking equipment installed for five years, the EB was at a higher level than after one month. Only the overall number of EB-positive samples decreased in the drinking equipment from one month to five years’ installation.

Although *S.* Derby was described as the most frequently found *Salmonella* serovar in the context of human salmonellosis originating from pork [21], a study from Ireland showed a high occurrence of *S. Typhimurium* and *S.* Manhattan in pig lairage pens, while *S.* Derby did not occur in a similar high frequency [71]. In the current study, *S.* Derby was the most common serovar found in the water distribution system, being isolated four times. *S. Typhimurium* was isolated only once, contrary to the studies proving it to be the main serovar [71]. *S. Typhimurium* has reportedly shown no ability to produce biofilm or can produce only very weak biofilm in a constantly flowing environment [72]. The findings in the drinking equipment installed for one and three months could possibly indicate briefly persistent biofilms produced by *Salmonella.* Nevertheless, *Salmonella* was found in the nipple drinkers, and so these could be infectious for pigs for the time the bacteria persist as or on biofilms. For these *Salmonella* serovars (*S.* Derby and *S. Typhimurium*), biofilm formation could be self-limiting and, compared with other serovars, could pose a lower risk of infection and cross-contamination, not considering the time they can still contaminate the abattoir and products while the biofilm is present. Other serovars that are more persistent, such as *S.* Agona or *S.* Montevideo, showed strong biofilm forming abilities in a condition simulating feed- and fishmeal factories. These serovars could also be able to form strong biofilms in a hydrodynamic environment and could be a continuous risk for contamination of the food chain [73,74,75].

As pathogenic *Y. enterocolitica* can form biofilm and was also found in the drinkers in this study, similar assumptions like those for *Salmonella* concerning infectivity and persistence could be made [76,77]. MRSA was not detected in any of the drinkers after they had been installed for three months, while at the other two sampling times, MRSA was found. Out of 33 lairage pens, only six were assessed in this study. The results do not mean that all the lairage pens were free of MRSA, as other drinkers could have harboured this pathogen. While *L. monocytogenes* is known to occur in pigs and can form biofilms, this pathogen was not detected at all.

Measures to reduce biofilm formation and load might be regular cleaning and disinfection of the water distribution system [78,79,80]. The abattoir in this study cleans the water distribution system only on the outside using the same procedure as is used for the lairage pens. During the day, the pens are rinsed with cold water and once a week are cleaned with detergent. Therefore, only the drinkers and lower pipe sections are roughly cleaned on the outside, but the inner surfaces of the water distribution system are not cleaned at all. Flushing of pipes and drinking equipment is inefficacious in totally eradicating biofilm, as the necessary pressure to clean pipes in water distribution systems of all biofilm is impossible to achieve [64]. Another measure could be coating, i.e., nanotechnological coating of the surfaces to prevent adhesion [81]. The practicability and effectiveness of these methods should be evaluated in further studies.

### 4.3. Distribution of Bacterial Content in the Drinkers

The examination of the drinkers revealed that retrograde growth in the drinking systems in lairage pens was present. The distribution and quantity of bacteria and pathogenic microorganisms in the drinkers and corresponding pipes varied. Numerous factors could influence the different bacteria occurrences and biofilm formation rates in the lairage pen drinkers included in this study.

One category of factors that might affect the biofilm formation rates and bacteria occurrences includes the pigs as a group and as individuals. On the first level, the origin of the pigs can be of importance. The abattoir where the samples were taken received pigs from different fattening farms, including pigs raised under organic production conditions. Each farm has its own management strategies and potentially, therefore, a different status concerning animal health. As a result, it is possible to have differently contaminated lairage pens, when one part of the lairage pens by chance holds pigs with higher bacterial loads, transferring the pathogens to the environment and, if the animals ingest water, to the drinking equipment [71]. Thus, the drinking system can be contaminated by development of biofilms and retrograde growth, as Vogels et al. (2020) [7] were able to show for piglet nurseries. Furthermore, it is important to consider the pigs as individual animals. Pigs can carry pathogens on their mucous membranes or other surfaces and transfer those pathogens into the environment of the lairage pens [33,37,82]. In any combination of an individual pig and the whole delivery group, it is possible that a part of one delivery group is more infected in comparison to others. By chance, a non-homogeneous distribution of infected or carrier pigs over the lairage pens used can be assumed, resulting in differing contamination rates of these used lairage pens and drinkers. Any non-regular occupancy of marginal or harder-to-reach-pens would also contribute to differing biofilm formation.

Another particularly important category of factors influencing the contamination of the lairage pens and drinkers might be the time spent in the lairage pens before slaughter. Logically, the longer the waiting period before slaughter, the greater the number of pathogens contaminating the surroundings from already infected or carrier pigs. The longer infected or carrier pigs stay in the lairage pens, the more they move, use enrichment materials, drink water and produce waste [83], which can eventually lead to more contaminated surfaces. If, as shown in this study, high bacteria levels and specific pathogens are present in/on the inner and outer surfaces of the drinkers, this could lead to the contamination of the pens. Finally, this could result in both the exchange of microorganisms, including pathogens, between pigs within the same delivery group, and the transfer of the microorganisms to the next batches of pigs from different farms. These next batches of pigs have direct contact with the contaminated drinkers. Furthermore, in the studied abattoir, the pigs are moved through the lairage pens with Drinkers A, C and F in the front of the lairage area to be sorted in the back of the lairage (pens with Drinkers B and E) (Figure 1). Pigs with faeces and other contamination on their legs, sides, backs and especially noses visibly can transfer these contaminations to drinkers and other lairage pen surfaces.

In this study, none of the previously described factors that could possibly influence the biofilm formation rates and bacteria occurrence were evaluated simultaneously with the duration for which the drinking equipment had been installed. To fully understand the extent of these factors further studies are necessary.

Aside from the pigs, environmental factors must be considered as possible influencing factors on different contamination rates and biofilm formation. The water distribution system used in the abattoir examined is of a branched kind, resulting in dead ends in the system and different distances the water must cover for it to get to the different lairage pens. Decreases in water pressure and water velocity could occur with longer distances from the main piping system. Both the dead ends and the distances could facilitate biofilm formation in the areas examined in this study [84,85]. Additionally, seasonal changes could affect biofilm formation [86], but in this study, air temperature data was not continuously collected. Instead, the air temperature was measured and recorded on sampling days. The highest temperature occurred when the drinking equipment installed for three months was sampled, with a lairage temperature of 18 °C and outdoor temperature of 10 °C.

Referring to Section 3.5 *Evaluation of the Drinkers*, the occurrence of biofilm formation should be evaluated with the sample types P7 and P8, as these demonstrate the actual stationary biofilm. Overall, Drinkers E and B showed the highest bacteria counts in the three installation durations, followed by Drinker D. The lowest bacteria counts were seen in Drinkers A and C. For the specific pathogens, Drinker D had the highest number of isolations of pathogenic microorganisms, followed by Drinker E. These drinkers have a more central location in the lairage and are closer to the start of the water distribution system. Drinkers A and C were, overall, the least contaminated drinkers, with the lowest bacteria counts. The distance to the start of the water distribution system was relatively long in Drinker A, where the overall lowest bacteria counts were seen. This disagrees with the previous consideration that a lower water velocity might increase the biofilm formation [84,85], because less biofilm is flushed out. This stands out especially when compared to Drinker B, which showed high bacteria counts and harboured specific pathogens, but, similarly to Drinker A, involved the longest distance to the start of the water distribution system. In another example, Drinker C, in a central position and a lairage pen through which pigs are moved, had lower bacteria counts and pathogens were detected less often here than in Drinker D, in a comparable central position. Consideration of other factors influencing the water distribution system is needed to understand the characteristics affecting biofilm formation.

The time spent in the lairage pens can influence the number of pathogens transferred to the surroundings and drinking equipment. It can also influence the number of pathogens ingested by pigs. The longer the waiting period before slaughter, the more it can be assumed that pigs get infected by pathogens from the surrounding area, including from drinkers. A study by Hurd et al. (2001) [5] proved that a time of two hours was sufficient for pigs to be infected by *Salmonella* from contaminated lairage pens. The pathogens were isolated from lymph nodes and ileum sections of acutely infected pigs. Although the time spent in the lairage pens is often less than two hours, a potential risk of infection can still be expected. In addition, *Y. enterocolitica* and *L. monocytogenes* accumulate in the pigs’ tonsils [31,87] and can, thus, pose a risk of cross-contamination when carcasses are split during the slaughter process. Higher infection rates with time are supported by the relationship with prolonged transportation times. Long transport increases the stress level of the animals, making them more prone to infections. Therefore, pigs that were transported longer have a higher risk of getting infected in the abattoir, even if they spent only a short time in the lairage [88,89,90].

### 4.4. Evaluation of the Comparability of Microbial Loads

A critical point in the evaluation of these results is the comparability of the microbial loads. The individual nipples have an unknown and undefinable surface area, which is why in the calculation, the expanse was counted as one, describing microbial loads on samples P3–P6 as CFU per nipple, not per cm^2^ [51]. Compared to the other sample types (excluding P1), which were calculated with a definite area, the results differed to a great extent. As an example, one single CFU in sample type P3–P6 resulted in a calculated value of 4.00 log CFU/nipple. The same number of CFU for the same dilution level for sample type P2 would result in a calculated value of 2.05 log CFU/cm^2^. This can dissemble analysis, indicating much higher contamination for the P3–P6 samples, even though the number of CFU at the same dilution levels are equal. Therefore, the sample type with the highest contamination rate regarding APC, EB and *Pseudomonas* spp. could not be ascertained for P2 or P3–P6. As for the specific pathogens, positive results were mainly obtained from the individual nipples (P3–P6), and these organisms were not evaluated quantitively.

## 5. Conclusions

To the best of our knowledge, this is the first study examining biofilm formation and occurrence of specific pathogens in the drinking water system of a pig abattoir lairage facility. In conclusion, this study demonstrates the occurrence of APC, EB, *Pseudomonas* spp., *Salmonella*, pathogenic *Y. enterocolitica* and MRSA in drinking nipples and pipes in lairage pens in a pig abattoir. All six drinkers examined after being installed for three different time durations showed repetitive findings of APC (97.2% of all samples), EB (27.8% of all samples) and *Pseudomonas* spp. (49.2% of all samples). While *Salmonella* and pathogenic *Y. enterocolitica* were only found in the drinkers after they had been installed for one month (*S.*: 9.5%; *Y.*: 7.1%) or three months (*S.*: 2.4%; *Y.*: 2.1%), MRSA was found after the drinkers had been installed for one month (7.1% of all samples) and five years (11.1% of all samples). Retrograde growth of biofilm was detectable to the upper pipe section of the drinking equipment, at least 88 cm from the nipples, close to the connection to the main water distribution system. Specific pathogens can likely contaminate the drinking equipment because they were found especially in samples from the nipple area that has direct animal contact. The results show biofilm and specific pathogens are present in the drinking system; both pathogens and detached biofilm can be ingested by pigs that have contact with the nipple drinkers or drink the water. This demonstrates a possible route for contamination in pig abattoirs. Even though pigs stay only a short time in lairage pens, infections are possible and should, therefore, be considered when measures for prevention of food chain contamination are reviewed. Thus, the water distribution systems in the lairage must be considered a source of infection for pigs and a potential risk for food hygiene and safety.

## Figures and Tables

**Table 1:** Overview of total aerobic plate count (APC), *Enterobacteriaceae* count (EB) and *Pseudomonas* spp. for the eight sample types examined per drinker (A–F) and for the three time durations the drinkers were installed.

	Sample Type		P1			P2			P3–P6			P7			P8			Total Count °		
		Time Installed	1 m	3 m	5 y	1 m	3 m	5 y	1 m	3 m	5 y	1 m	3 m	5 y	1 m	3 m	5 y	1 m	3 m	5 y
	Drinker																			
APC	A		6.10	4.00	4.88	6.18	6.71	5.65	7.26	7.51	6.97	1.78	3.94	3.68	0.85	1.43	0.00	48/48 (100%)	47/47 (100%)	45/48 (93.8%)
	B		4.21	5.09	5.42	6.19	6.92	5.98	7.73	7.63	7.81	2.39	3.47	3.37	2.01	4.63	1.85			
	C		4.29	4.44	4.50	6.69	5.82	5.75	7.24	7.59	7.37	2.86	3.54	2.65	1.03	2.59	0.00			
	D		3.96	4.11	5.58	6.95	7.08	5.61	7.71	7.66	7.54	2.27	3.62	4.33	1.53	/*	1.85			
	E		4.43	4.47	5.08	7.23	7.22	6.04	7.73	7.68	7.51	2.87	3.72	2.78	1.46	2.10	0.00			
	F		4.01	4.88	4.62	6.94	7.02	6.20	7.89	7.90	7.62	1.69	2.84	4.08	2.30	2.09	1.85			
EB	A		0.00	0.00	0.00	2.31	1.35	0.00	0.00	0.75	0.00	0.00	0.00	0.00	0.00	0.00	0.00	15/48 (31.3%)	13/48 (27.1%)	12/48 (25.0%)
	B		0.00	0.00	0.00	0.00	1.93	0.00	0.00	0.75	3.00	0.00	0.00	0.00	0.00	0.00	0.00			
	C		0.00	0.00	0.00	0.00	0.00	0.00	2.41	0.90	3.31	0.00	0.00	0.00	0.00	0.00	0.00			
	D		0.00	0.00	0.00	1.35	1.35	3.85	2.70	3.15	1.43	0.00	0.00	0.00	0.00	0.00	0.00			
	E		0.00	0.00	0.00	1.60	1.05	0.00	1.62	0.00	2.26	0.00	0.00	0.00	0.00	0.00	0.00			
	F		0.00	0.00	0.00	1.05	1.35	0.00	3.34	0.75	2.00	0.00	0.00	000	0.00	0.00	0.00			
*Pseudomonas* spp.	A		-	-	-	1.99	2.88	0.00	1.85	2.78	0.75	0.00	0.00	0.00	0.00	0.00	0.00	24/42 (57.0%)	25/42 (59.5%)	13/42 (31.0%)
	B		-	-	-	0.00	2.23	0.00	2.18	4.20	0.83	0.00	0.00	0.00	0.00	0.00	0.00			
	C		-	-	-	3.09	3.56	0.75	3.79	4.91	1.69	0.00	0.00	0.00	0.00	0.00	0.00			
	D		-	-	-	2.01	2.29	3.33	3.93	2.88	2.67	0.00	0.00	0.00	0.00	0.00	0.00			
	E		-	-	-	3.33	1.35	0.00	3.74	0.88	2.18	0.00	0.00	0.00	0.00	0.00	0.00			
	F		-	-	-	2.75	3.09	0.00	4.53	5.02	1.67	0.00	0.00	0.00	0.00	0.00	0.00			

P1 = water sample in log CFU/mL; P2 = pooled swab sample from all four nipple drinkers in log CFU/cm^2^; P3–P6 = mean value of the four individual nipple samples in log CFU/nipple; P7 = swab sample of the lower pipe section in log CFU/cm^2^; P8 = swab sample of the upper pipe section in log CFU/cm^2^; 1 m = one month installed drinking equipment; 3 m = three months installed drinking equipment; 5 y = five years installed drinking equipment; APC = total aerobic plate count, EB = *Enterobacteriaceae* count;—sample was not tested for *Pseudomonas* spp.; * the sample could not be examined; ° the total count describes all the samples with evaluable bacteria counts, regardless of the quantity.

**Table 2:** Overview of *Salmonella*, *Listeria monocytogenes,* pathogenic *Yersinia enterocolitica* and methicillin-resistant *Staphylococcus aureus* for the seven sample types examined per drinker (A–F) and for the three time durations the drinkers were installed.

	Sample Type		P2			P3–P6			P7			P8			Total Count		
Pathogen		Time Installed	1 m	3 m	5 y	1 m	3 m	5 y	1 m	3 m	5 y	1 m	3 m	5 y	1 m	3 m	5 y
	Drinker																
*Salmonella*	A		-	-	-	-	-	-	-	-	-	-	-	-	4/42 9.5%	1/42 2.4%	0/42 0.0%
	B		-	-	-	-	-	-	-	-	-	-	-	-			
	C		-	-	-	-	-	-	-	-	-	-	-	-			
	D		-	-	-	-	+ °	-	-	-	-	-	-	-			
	E		+ *	-	-	+++ *	-	-	-	-	-	-	-	-			
	F		-	-	-	-	-	-	-	-	-	-	-	-			
*Listeria* *monocytogenes*	A–F		All samples were negative.												-	-	-
Pathogenic *Yersinia* *enterocolitica*	A		-	+	-	-	-	-	-	-	-	-	-	-	3/42 7.1%	1/42 2.4%	0/42 0.0%
	B		-	-	-	-	-	-	-	-	-	-	-	-			
	C		-	-	-	-	-	-	-	-	-	-	-	-			
	D		+	-	-	++	-	-	-	-	-	-	-	-			
	E		-	-	-	-	-	-	-	-	-	-	-	-			
	F		-	-	-	-	-	-	-	-	-	-	-	-			
Methicillin- resistant *Staphylococcus aureus*	A		-	-	-	-	-	-	-	-	-	-	-	-	3/42 7.1%	0/42 0.0%	11/42 26.2%
	B		-	-	-	+	-	++++ ^1^	-	-	-	-	-	-			
	C		-	-	+	-	-	+++ ^2^	-	-	-	-	-	-			
	D		-	-	-	+	-	-	-	-	-	-	-	-			
	E		-	-	-	-	-	+	-	-	-	-	-	-			
	F		-	-	+	+	-	+	-	-	-	-	-	-			

P2 = pooled swab sample from all four nipples; P3–P6 = incorporated results from the four individual nipple samples; P7 = swab sample of the lower pipe section; P8 = swab sample of the upper pipe section; 1 m = drinking equipment installed for one month; 3 m = drinking equipment installed for three months; 5 y = drinking equipment installed for five years;—shows the samples which were negative for the specific pathogens; + shows the samples which were positive for the specific pathogens; ^1^ all four samples were positive; ^2^ three of the four samples were positive; * *S.* Derby identified; ° *S. Typhimurium* identified.

## Data Availability

The data presented in this study are available in the supplementary materials in Table S1—Results of total aerobic plate count and *Enterobacteriaceae;* Table S2—Results of the specific pathogens.

## Supplementary Materials

The following supporting information can be downloaded at: https://www.mdpi.com/article/10.3390/microorganisms11102554/s1; Table S1—Results of total aerobic plate count and *Enterobacteriaceae;* Table S2—Results of the specific pathogens.

## Appendix A

**Table A1:** Results of Kruskal-Wallis (KW) test to detect differences in bacteria count in sample types considering the three time durations together.

		KW Statistic	*p*-Value
	Sample Type		
APC	P1	5.1720	0.0753
	P2	8.5029	0.0142
	P3–P6	1.7193	0.4233
	P7	7.9415	0.0189
	P8	5.6911	0.0581
EB	P1	0.0000	1.0000
	P2	2.7134	0.2575
	P3–P6	1.5161	0.4686
	P7	0.0000	1.0000
	P8	0.0000	1.0000
*Pseudomonas* spp.	P2	4.8738	0.0874
	P3–P6	7.2209	0.0270
	P7	0.0000	1.0000
	P8	0.0000	1.0000

P1 = water sample in log CFU/mL; P2 = pooled swab sample from all four nipple drinkers in log CFU/cm^2^; P3–P6 = mean value of the four individual nipple samples in log CFU/nipple; P7 = swab sample of the lower pipe section in log CFU/cm^2^; P8 = swab sample of the upper pipe section in log CFU/cm^2^; APC—total aerobic plate count, EB—*Enterobacteriaceae* count.
